# The Dual Role of TRIM7 in Viral Infections

**DOI:** 10.3390/v16081285

**Published:** 2024-08-12

**Authors:** Maria Gonzalez-Orozco, Carlos A. Rodriguez-Salazar, Maria I. Giraldo

**Affiliations:** 1 Department of Microbiology and Immunology, University of Texas Medical Branch, Galveston, TX 77555, USA; maridgon@utmb.edu (M.G.-O.); crodriguez4@cue.edu.co (C.A.R.-S.); 2 Molecular Biology and Virology Laboratory, Faculty of Medicine and Health Sciences, Corporación Universitaria Empresarial Alexander von Humboldt, Armenia 630003, Colombia

**Keywords:** TRIM7, ubiquitination, antiviral response, viral infections

## Abstract

The E3 ubiquitin ligase TRIM7 is known to have dual roles during viral infections. Like other TRIM proteins, TRIM7 can regulate the IFN pathway via the regulation of the cytosolic receptors RIG-I or MDA-5, which promote the production of type I interferons (IFN-I) and antiviral immune responses. Alternatively, under certain infectious conditions, TRIM7 can negatively regulate IFN-I signaling, resulting in increased virus replication. A growing body of evidence has also shown that TRIM7 can, in some cases, ubiquitinate viral proteins to promote viral replication and pathogenesis, while in other cases it can promote degradation of viral proteins through the proteasome, reducing virus infection. TRIM7 can also regulate the host inflammatory response and modulate the production of inflammatory cytokines, which can lead to detrimental inflammation. TRIM7 can also protect the host during infection by reducing cellular apoptosis. Here, we discuss the multiple functions of TRIM7 during viral infections and its potential as a therapeutic target.

## 1. Introduction

Post-translational modifications (PTMs) of proteins, such as ubiquitination, are necessary to regulate the function and stability of a given protein [1]. The ubiquitination process consists of the addition of the small protein ubiquitin (Ub) to the lysine (K) residue of a target protein [2]. Ub itself possesses 7 lysine residues (K6, K11, K27, K29, K33, K48, and K63) and the α amino group of the N-terminal methionine (Met1), where another Ub can be covalently attached to form polyubiquitin chains [3,4,5]. Three enzymes are necessary for the ubiquitination process: the E1-activating enzyme, which activates Ub in an ATP-dependent reaction, the E2-conjugating enzyme, which catalyzes the transfer of the Ub to the target protein, and the E3 ubiquitin ligases which determines the target of ubiquitination [6,7]. The E3-Ub ligases are classified into three types: RING finger, HECT, and RBR types [8]. The RING finger type binds to the E2-conjugating enzyme and the substrate protein, facilitating the transfer of Ub from the E2 to the target protein [9]. This group includes the tripartite motif-containing (TRIM) proteins that have been shown to play dual roles during viral infections [10]. Multiple studies have shown that TRIMs have antiviral activity by promoting Type I Interferon (IFN-I) responses during viral infections or by targeting viral proteins for degradation [2,11,12,13,14,15,16]. However, during some viral infections, TRIM proteins can negatively affect the antiviral response or directly promote virus infection by modifying viral proteins [17,18,19].

The structure of the TRIM proteins encompasses the conserved RBCC domain, which includes a RING E3 ligase domain (R), one or two B-box motifs (B), and a coiled-coil domain [20]. The RING domain is responsible for the interaction with the E2 Ub-conjugating enzymes, and the CC domain promotes oligomerization [20,21], while the C-terminal varies between the different TRIM proteins. The most common domain found is the PRY-SPRY (B30.2), either in combination or individually, and this domain is involved in substrate recognition [10,20]. In this work, we will focus on the E3-Ub ligase TRIM7 and discuss its dual roles during viral infections and its potential as a therapeutic target.

## 2. TRIM7 Structure and Isoforms

**Figure 1:**
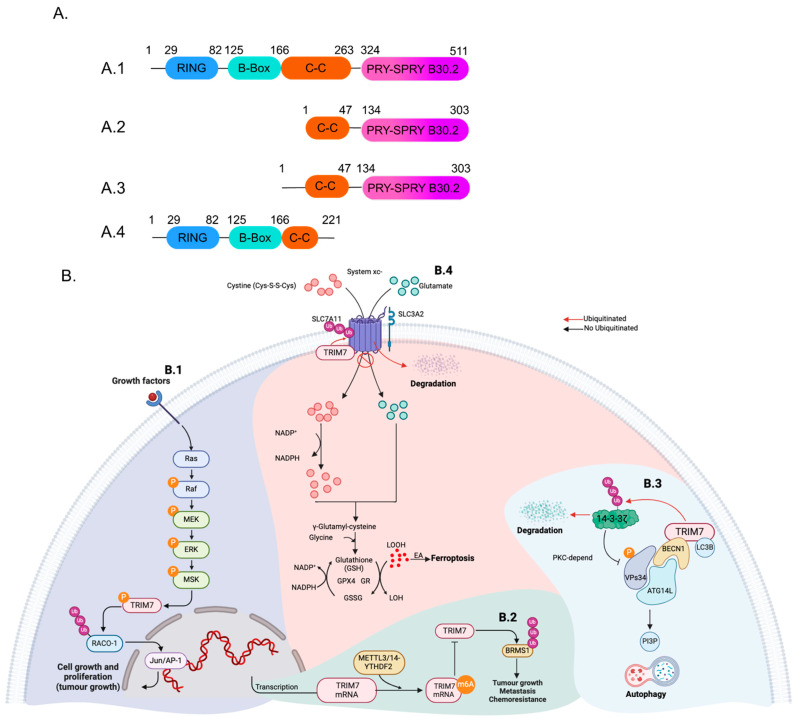
The pro- and anti-tumoral functions of TRIM7. (**A**) Depicts the predicted protein architecture encoded by the GNIP (TRIM7) gene in cartoon form. The numbers indicate the approximate demarcation of the different domains for (**A.1**) GNIP1, (**A.2**) GNIP2, (**A.3**) GNIP3, and (**A.4**) TRIM7 short form. (**B.1**) The c-Jun/AP-1 transcription factor is crucial for proliferation and apoptosis. MSK1 phosphorylates TRIM7 in response to direct activation by the Ras-Raf-MEK-ERK pathway. This modification stimulates TRIM7 and mediates Lys63-linked ubiquitination of RACO-1, leading to RACO-1 protein stabilization. Consequently, TRIM7 depletion reduces RACO-1 levels and RACO-1-dependent gene expression. (**B.2**) Modifying TRIM7 mRNA with N6-methyladenosine (m6A) through METTL3/14-YTHDF2 decreases its expression. This regulation enables TRIM7 to control the migration and invasiveness of osteosarcoma cells by ubiquitinating the breast cancer metastasis suppressor (BRMS1). (**B.3**) TRIM7 can regulate autophagy. Biochemically, GNIP1 binds to BECN1 and LC3B, inducing autophagy by promoting the formation of autophagic protein complexes. Additionally, TRIM7 promotes autophagy progression by mediating the K48-linked ubiquitination of 14-3-3ζ, a negative regulator of autophagy. (**B.4**) Ferroptosis is induced by the excessive accumulation of lipid hydroperoxides in the cellular membrane. GPX4 regulates lipid peroxidation using GSH to reduce lipid hydroperoxides (LOOH) to lipid alcohols (LOH), suppressing ferroptosis. GSH is oxidized to GSSG in this process. When SLC7A11 is ubiquitinated BY TRIM7, the system xc- is truncated, accumulating LOOH and subsequent ferroptosis, inhibiting gastric cancer progression. GR: glutathione reductase, GPX4: glutathione peroxidase 4, GSH: reduced glutathione, GSSG: oxidized glutathione (glutathione disulfide), LOOH: lipid hydroperoxide, LOH: lipid alcohol, EA: excessive accumulation. Black arrows indicate positive activation of the pathway, and red arrows indicate negative regulation of the pathway by promoting degradation of the protein. Figure was created with BioRender.com.

Human TRIM7, also known as RNF90, has an approximate molecular weight of 56 kDa. It was initially identified in a yeast two-hybrid system as a protein that interacts with and activates glycogenin-1 (GNIP) [22]. The structure of TRIM7 consists of the conserved RBCC region, which includes a RING domain, a B-Box domain, and a CC domain. The C-terminal region contains the PRY-SPRY domain (Figure 1A).

The *GNIP1* gene (also called *TRIM7*), encodes four spliced isoforms TRIM7/GNIP1 (referred to here as simply TRIM7), GNIP2, GNIP3, and a short form of TRIM7 [23]. Out of these four isoforms, only TRIM7/GNP1 and the short form possess the RING domain typical of the TRIM proteins [23] (Figure 1A). On the other hand, GNP2 and GNP3 are truncated isoforms with a shorter N-terminal domain that includes the CC domain and the conserved C-terminal domain B30.2, which is responsible for the binding with glycogenin-1 (Figure 1A) [23,24]. It is important to note that different names have been used in the literature for different isoforms disregarding the conventional concept that a canonical TRIM should contain the RBCC domain. In some cases, GNIP1 has been used to refer to the full-length gene containing the tripartite motif while TRIM7 is a shorter isoform [25]. Here, we adhere to the common nomenclature, and we refer to TRIM7 as the long isoform containing the RBCC-SPRY domains.

TRIM7 is expressed in several organs in mice, it has been observed that the brain, kidney, liver, lung, heart, muscle, testis, and uterus have high expression of TRIM7 [26,27]. At the cellular level, innate cells such as B cells, macrophages, and dendritic cells (DCs) express high levels of this E3 ligase [27]. TRIM7 is also expressed in human PBMCs, and its expression was downregulated in sepsis patients [28].

The truncated form of TRIM7 has been linked to the reduction of tumor growth by promoting apoptosis [25].

## 3. TRIM7 in Non-Viral Conditions

TRIM7 has been shown to regulate various cellular processes, including autophagy, cell death, migration, and invasiveness of tumor cells. After activation of the JNK and Ras/MAPK signaling pathway by cell growth factors, TRIM7 is phosphorylated at S107. This phosphorylation enables TRIM7 to ubiquitinate and stabilize RACO-1, allowing the activation of c-Jun/AP-1 to promote the transcription of target genes important in promoting cell survival and proliferation, which increases lung tumor growth progression [29] (Figure 1B). Moreover, TRIM7 was found to be expressed at higher levels in tissues of patients with osteosarcoma, and its increased expression correlates with poor prognosis in these patients; mechanistically, TRIM7 positively regulates cell migration and invasiveness of osteosarcoma cells by ubiquitinating the breast cancer metastasis suppressor (BRMS1) at the lysine K184 [30]. The expression of TRIM7 is regulated at the mRNA level by the N6-methyladenosine (m6A) modification which is mediated by METTL3 and METTL14 (Figure 1B). This modification affects the stability of the mRNA leading to reduced levels of TRIM7 at the protein level. It was observed that samples from osteosarcoma patients showed reduced m6A modification of TRIM7 mRNA, thereby increasing the levels of TRIM7 protein and its pro-tumoral functions [30].

TRIM7 was also shown to induce cancer progression in lung cancer cells by promoting the degradation of 14-3-3 ζ (a negative regulator of Vsp34) to trigger autophagy (Figure 1B). Additionally, TRIM7 can interact with Beclin-1 and LC3B to induce the formation of the autophagosomes and promote autophagy, which can lead to the proliferation and migration of lung cancer cells [31].

Conversely, other studies have demonstrated that TRIM7 is downregulated in samples from gastric cancer (GC) patients. This downregulation is significant as TRIM7 acts as a tumor suppressor, inhibiting the proliferation of GC cells and inducing ferroptosis [32] (Figure 1B). The induction of ferroptosis occurs by the K48 ubiquitination and subsequent degradation of the solute carrier family 7 member 11 (SLC7A11), and is a particularly intriguing aspect of TRIM7’s role in gastric cancer cells [32]. Additionally, TRIM7 negatively regulates the Src-mTORC1-S6K axis in hepatocellular carcinoma cells and ubiquitinates the Src kinase in a K48 manner to promote its degradation. Lower levels of Src kinase reduced activation of both mTORC1 and S6K, leading to reduced proliferation and invasiveness of the hepatocellular carcinoma cells [33]. There is only one report describing the role of TRIM7 in bacterial infections; during *L. monocytogenes* infection, TRIM7 reduces bacteria burden in mice by promoting K63 ubiquitination of ATG7 and induces autophagy [34].

All of these studies highlight the diverse functions of TRIM7 within the cell, as it regulates essential cellular processes. However, due to it belonging to the TRIM family, extensive research has been conducted on its role in viral infections.

## 4. TRIM7 Regulates the Antiviral Innate Immune Response

The innate immune antiviral response is induced in the cell after stimulation of pattern recognition receptors (PRRs) that detect the pathogen-associated molecular patterns (PAMPs) of the pathogens [35]. In the case of viruses, the viral nucleic acids are the main PAMP recognized, activating the signaling pathways to induce the transcription of genes encoding pro-inflammatory cytokines and IFNs required to inhibit virus replication [36].

Some of the most well-characterized PRRs responsible for virus recognition are the endosomal Toll-like receptors (TLRs) TLR3, TLR7, TLR8, and TLR9, as well as the retinoic-acid-inducible gene I (RIG-I)-like receptors (RLRs) expressed in the cytoplasm, such as RIG-I, melanoma differentiation-associated gene 5 (MDA5), and laboratory of genetics and physiology 2 (LGP2) [35,36].

**Figure 2:**
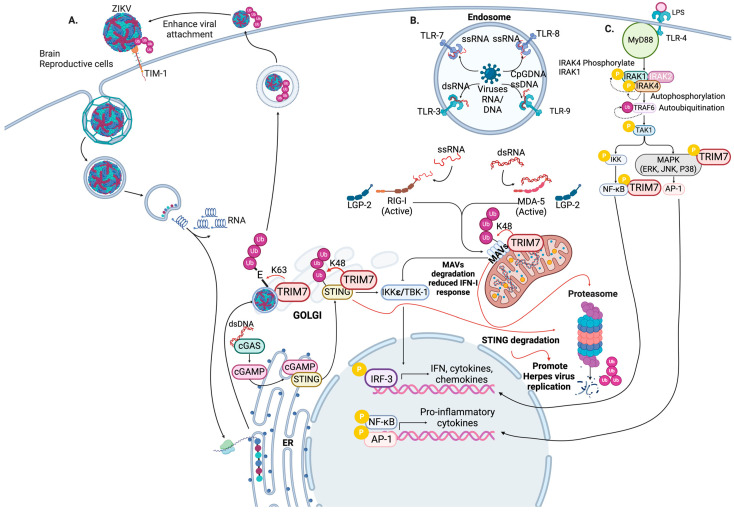
Proviral and antiviral roles of TRIM7. (**A**) During ZIKV infection, viral entry occurs after viral attachment and endocytosis that leads to clathrin-mediated endocytosis, followed by uncoating of the viral genome, and after viral genome replication, the viral polyprotein is generated; this leads to viral assembly in the ER where E protein is ubiquitinated in a K63 manner by TRIM7, the virus is then matured and is released from the cells. The ubiquitination of E protein mediated by TRIM7 enhances the binding of the virus with its receptor TIM-1, increasing viral replication in the brain and reproductive cells. (**B**) TRIM7 regulates innate immune receptors. Upon recognition of PAMPs, pattern recognition receptors such as endosomal Toll-like receptors (TLRs 3, 4, 7–9) and cytosolic RIG-I-like receptors initiate signaling pathways to induce the production of pro-inflammatory cytokines and interferons (IFNs), TRIM7 negatively regulates major cytosolic receptors RIG-I and MDA-5 by inducing K48-linked ubiquitination of the adaptor protein MAVs, leading to its degradation via the proteasome, thus inhibiting IFN-I production. Similarly, TRIM7 ubiquitinates the DNA receptor STING in a K48-linked manner, resulting in proteasomal degradation and reduced IFN-I production, facilitating increased replication of DNA viruses such as HSV-1. (**C**) TRIM7 also positively regulates the inflammatory response following TLR4 activation by LPS. TRIM7 expression is crucial for inducing the phosphorylation of ERK1/2, p38, JNK1/2, IKKα/β, and IRF3, leading to the activation of signaling pathways for the production of cytokines such as IL-6, TNF, and IFN-β in macrophages. PAMPs: pathogen-associated molecular patterns, TLRs: Toll-like receptors, MAVs: mitochondrial antiviral-signaling protein, STING: stimulator of interferon genes, LPS: lipopolysaccharide, ERK: extracellular signal-regulated kinase, JNK: c-Jun N-terminal kinase, IKK: IκB kinase, IRF: interferon regulatory factor, IL: interleukin, TNF: tumor necrosis factor. Black arrows indicate positive activation of the pathway, and red arrows indicate that TRIM7 promotes the degradation of the protein. Figure was created with BioRender.com.

TRIM7 negatively affects the signaling pathway of the major cytosolic receptors RIG-I and MDA-5 by inducing K48 ubiquitination of the adaptor protein mitochondrial antiviral-signaling protein (MAVs) leading to its degradation through proteasome and inhibiting the production of IFN-I [37] (Figure 2B). In a similar mechanism, TRIM7 has been shown to ubiquitinate the DNA receptor stimulator of interferon genes (STING) in a K48-linked manner, inducing its proteasomal degradation. Reduced levels of STING result in decreased IFN-I production and increased replication of DNA viruses such as HSV-1 [38] (Figure 2B). TRIM7 can also interact with RIG-I and MDA-5. Increasing concentrations of TRIM7 reduced the IFN-β promoter activity induced by MDA-5, suggesting that TRIM7 downregulates IFN-β signaling production through its interaction with MDA-5. However, further studies are necessary to elucidate the mechanism of action [39]. On the other hand, other studies suggest that TRIM7 can positively regulate the IFN-I response induced by RIG-I or MDA-5 activation by interacting with MAVs, thus increasing levels of IFN-β and limiting the replication of encephalomyocarditis virus (EMCV) [40]. TRIM7 knockout placental JEG-3 cells also show reduced IFN-β induction when stimulated with the double-stranded RNA mimic poly I:C [26].

TRIM7 positively regulates the inflammatory response after TLR4 activation by LPS. Further, TRIM7 expression is crucial for inducing the phosphorylation of ERK1/2, p38, JNK1/2, IKKα/β, and IRF3, leading to downstream signaling and production of cytokines such as IL-6, TNF, and IFN-β in macrophages [27] (Figure 2C).

## 5. Proviral Role of TRIM7 during Virus Replication

TRIM7 has also been reported to promote virus pathogenesis showing proviral activity by enhancing virus replication. Our recent findings suggest that the envelope (E) protein of Zika virus (ZIKV) undergoes K63-linked polyubiquitination by TRIM7, which enhances its replication in the brain and reproductive tissues [26] (Figure 2A). Evidence indicates that flaviviruses contain ubiquitinated viral proteins in the infectious virion that promote interactions with receptors outside cells. For instance, a lysine residue (K38) on the E protein of ZIKV has been found to play a direct role in viral entry. This residue is also conserved in other members of the flaviviridae family, such as dengue virus (DENV2), West Nile virus (WNV), and yellow fever virus (YFV). During infections, the replication of a recombinant mutant E-K38R of ZIKV (ZIKV E-K38R) was significantly attenuated in a cell-type-specific manner [26]. Additionally, TRIM7 was found to promote the ubiquitination of ZIKV E in vitro and in cells. In addition, *Trim7^−/−^* mice showed reduced virus replication, especially in the brain and reproductive tissues of infected mice. Also, a proportion of infectious viral particles contained ubiquitinated E. The E-K38 residue was found to be critical for interactions between E and the cellular receptor TIM-1 [26] (Figure 2A). This interaction enhanced the virus’s entry, replication, and pathogenesis. Therefore, ubiquitination of E promotes virus entry and is crucial in determining the virus’s tissue tropism. Further evidence suggests that attachment and replication of the virus can be improved by ubiquitination of E, as demonstrated by neutralization assays using an anti-K63-linked-polyubiqutin antibody in tissue culture and in vivo [26]. It is currently unknown where in the cell the process of E ubiquitination takes place and how the ubiquitinated E is integrated into the virion; however fractionation and confocal analyses suggest this occurs in or around the Golgi compartment. In addition to the K38 site, the E protein is also ubiquitinated on the K281 residue, which could impact the fusion or uncoating steps [26,41]. The exact role of this ubiquitination in viral replication is still uncertain. These insights could potentially aid in developing more effective treatment strategies and ultimately help mitigate the pathogenesis associated with ZIKV infection.

## 6. Antiviral Roles of TRIM7

### 6.1. TRIM7 in Enteric Virus Infection

**Figure 3:**
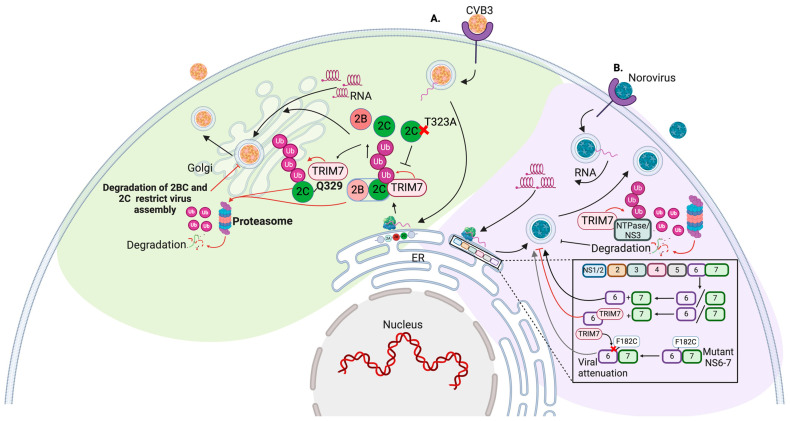
The antiviral role of TRIM7 during enteroviruses replication. (**A**) TRIM7 recognizes and ubiquitinates the viral protein 2BC of coxsackievirus (CVB3), a precursor of the 2C protein, TRIM7 recognized the 2C portion of the protein, specifically the glutamine Q329 leading to its ubiquitination and degradation of the protein through proteasome reducing the CVB3 replication. After multiple passages, the pressure exerted by TRIM7 led to a mutation in the protein 2C on position T323, a change for an alanine to avoid the restriction mediated by TIRM7. (**B**) In norovirus infection, TRIM7 recognized the glutamine-end motif of the NTPAse/NS6 protein leading to ubiquitination and degradation of this one reducing virus replication. TRIM7 can also bind to the viral protease NS6 but not to the NS6-7 precursor protein. Through a mechanism not described yet, this interaction regulates viral replication; however, in a way to escape TRIM7 recognition, norovirus is able to induce a mutation in the position F182 avoiding the targeting by TRIM7 and allowing the virus to replicate. Black arrows indicate positive activation of the pathway, and red arrows indicate that TRIM7 promotes degradation of the protein, inhibitory red arrows indicate the inhibitory effect mediated by TRIM7-induced ubiquitination. Figure was created with BioRender.com.

TRIM7 exhibits antiviral activity against several human enterovirus types, including EV71 (type A), CVB3 and E11 (type B), and PV (type C), as well as the non-human enterovirus mengovirus (MenV) [42]. High-throughput screening analysis of 118 E3-Ub ligases identified TRIM7 as a potent inhibitor of coxsackievirus B3 (CVB3) infection [42]. The antiviral effects of TRIM7 are primarily mediated by ubiquitination and proteasomal degradation of the viral protein 2BC of CVB3 [42]. 2BC protein is the proteolytic precursor of 2C, an ATP/helicase, and an essential protein for viral RNA replication [43] (Figure 3A). TRIM7 recognized 2C by its PRY-SPY B30.2 specifically at the C-terminal glutamine at position 329 of 2C [44,45] (Figure 3A). The recognition of this C-terminal glutamine by TRIM7 targets the protein to proteasome-dependent degradation and represents the antiviral activity mediated by TRIM7 [44,45]. Moreover, the C3 protease (C3pro) of enterovirus and norovirus is responsible for the cleavage of the polyproteins. It can cleave proteins after a glutamine, producing a C-terminal glutamine that can be recognized by TRIM7 and acts as a C-dregon motif [44,46].

Genome-wide CRISPR activation screening during murine norovirus (MNV) infection found that TRIM7 was able to block the virus at the post-entry stage of the virus cycle [47,48]. This inhibition is dependent on the ability of TRIM7 to interact with NS6 (or 3C-like protease) [47]. Also, sequence alignment analysis showed that the NTPase protein from human and murine norovirus have the glutamine-end motif recognized by TRIM7, and functional analysis revealed that TRIM7 interacts and ubiquitinates norovirus NTPase to restrict virus replication [44] (Figure 3B).

It is interesting that C3pro not only cleaves viral proteins but also targets the host proteins. This quality represents an advantageous strategy for the virus to evade recognition by C3pro. Studies have shown that C3pro can cleave TRIM7 at glutamine 24 (Q24), rendering cleaved TRIM7 unable to exert its antiviral activity, resulting in increased virus replication [49]. Interestingly, this group also demonstrated that the cleavage residue Q24 is conserved in mammals except for four marsupials that have a histidine 24 (H24), making them resistant to cleavage by 3Cpro. However, an alternate splice site results in the insertion of an exon in the marsupial TRIM7, which contains a distinct 3Cpro cleavage site at Q338 [49].

#### TRIM7 Antiviral Pressure Promotes the Emergence of Viral Variants

TRIM7 is a potent inhibitor of enteroviruses and norovirus infection. Given the pressure exerted by TRIM7; enteroviruses have developed several mechanisms to overcome its activity. It has been reported that after several passages of CVB3, the new viruses acquired a point mutation in the protein 2C (target of TRIM7) in the residue T323 changed by an alanine (T323A). This mutation confers resistance to TRIM7 and has a replication advantage over the WT strain [42] (Figure 3A).

In this same line, multiple passages of MNV in cells overexpressing TRIM7 showed that by passage 3 or 4, there were populations of viruses that were resistant to TRIM7 antiviral activity. Deep sequencing of these populations showed mutations between the cleavage site of NS6-NS7 polyprotein. Specifically, the amino acid at position 182, which is conserved among MNV strains and usually a phenylalanine, was mutated to a cysteine, serine, or valine (Figure 3B). These changes provided resistance to TRIM7 activity and allowed norovirus to evade recognition and replicate [48].

These studies show that TRIM7 represents an important restriction factor for enteric viruses by targeting the proteins for degradation. However, they also demonstrate the ability of enteroviruses and noroviruses to evolve and overcome this recognition and restriction, potentially leading to the generation of more pathogenic strains that can evade immune recognition.

### 6.2. The Role of TRIM7 during SARS-CoV-2 Infection

**Figure 4:**
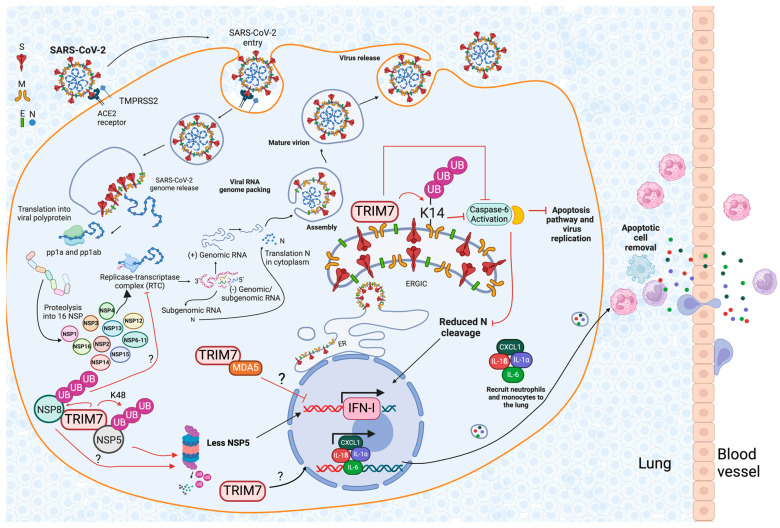
TRIM7 during SARS-CoV-2 infection. SARS-CoV-2 is recognized by ACE-2 receptor; following entry and uncoating of its genetic material, it starts replication and transcription of the proteins, and the polyprotein is cleaved into 16 non-structural proteins (NSPs) and 4 structural proteins. Then the RNA is packaged, and the viral particle is assembled to generate a mature virus. Non-structural proteins (NSPs) of SARS-CoV-2 and the structural membrane (M) protein possess a glutamine-end motif recognized by TRIM7. Specifically, NSP5 and NSP8 interact with TRIM7, leading to K48-linked ubiquitination and subsequent degradation of these proteins, thereby enhancing IFN-I production and reducing virus replication. TRIM7’s interaction with the M protein results in ubiquitination at the lysine 14 residue, which restricts apoptosis induced by M. Additionally, TRIM7 negatively regulates caspase-6 activation, preventing the cleavage of the nucleocapsid (N) protein thereby restricting apoptosis. TRIM7 regulates the inflammatory response during SARS-CoV-2 infection by inducing the production of pro-inflammatory cytokines IL-6, IL-1β, and IL-1α and the chemokine CXCL1 leading to the recruitment of monocytes and neutrophils to the infected lungs to help promote clearance of apoptotic cells and tissue repair. Black arrows indicate positive activation of the pathway, red arrows indicate that TRIM7 promotes degradation of the protein, inhibitory red arrows indicate the inhibitory effect mediated by TRIM7-induced ubiquitination, and question marks indicate that the effect is not confirmed experimentally but is suggested by the literature or downstream signaling elements have not been dissected yet. Figure was created with BioRender.com.

Recently, a study identified proteins from SARS-CoV-2 that have the glutamine-end motif and can potentially be recognized by TRIM7. The sequence alignment analysis showed that most of the non-structural protein (NSP) of SARS-CoV-2 as well as the structural protein membrane (M) have this glutamine-end motif [44]. Their work showed that NSP5 and NSP8 interact with TRIM7, resulting in K48-linked ubiquitination, which causes the degradation of these proteins [44] (Figure 4). NSP5 is an important inhibitor of IFN-I production [50,51,52,53,54], therefore reduced levels of NSP5 mediated by its degradation through proteasome resulted in increased levels of IFN-I. Thus, it was suggested that TRIM7 could have antiviral effects during SARS-CoV-2 infection in part by blocking virus antagonism.

This study, however, did not elucidate the role of the interaction between TRIM7 and M protein. Previous mass spectrometry analysis of SARS-CoV-2 viral proteins showed that TRIM7 can interact with M protein [55]. Our recent study has shown that TRIM7 exhibits antiviral activity against SARS-CoV-2 through multiple mechanisms. We found that TRIM7 regulates the cellular innate immune response during SARS-CoV-2 infection, promoting the recruitment of monocytes and neutrophils to the lung mediated by the induction of the chemokine CXCL1 (Figure 4), a major neutrophil chemoattractant [39]. In addition, TRIM7-deficient mice also exhibited reduced production of pro-inflammatory cytokines compared with the WT mice suggesting that TRIM7 is important for the production of pro-inflammatory cytokines IL-6, IL-1β, and IL-1α [39] (Figure 4). These effects may be mediated by the activation of AKT, as TRIM7 promoted the phosphorylation of AKT after TNF stimulation. Other pathways, like IKK-β activation, are not affected in the absence of TRIM7 [39].

TRIM7 can interact with and ubiquitinate the SARS-CoV-2 M protein on the K14 residue, and this ubiquitination restricts apoptosis [39]. The mutant virus unable to be ubiquitinated on M-K14 and K-15 residues showed reduced replication most likely due to the importance of K-15 ubiquitination and virus budding [56], but also showed increased apoptosis and higher pathology than WT virus apoptosis during virus infection. During coronavirus infection, the apoptosis pathway, especially caspase-6 activation, was shown to promote virus replication by cleavage of the nucleocapsid (N) protein; these cleavage products of N can inhibit the IFN-I response leading to increased virus replication [57]. Interestingly, TRIM7 can negatively regulate caspase-6 activation, inhibiting the cleavage of N and restricting the induction of apoptosis [39] (Figure 4). However, TRIM7 antiviral activity via inhibition of caspase-6 and apoptosis does not seem to be mediated by IFN-I [39], suggesting that the proviral effects of caspase-6 may be independent of cleavage of N or may have both IFN-dependent and independent effects. Apart from its degradative functions, TRIM7 can also regulate the inflammatory response and inhibit SARS-CoV-2 replication.

Importantly, longitudinal analysis of SARS-CoV-2 sequences of the so-called variants of concern (VOCs) during the pandemic indicated that mutations on M-K14 appeared, albeit in low proportion, in early variants [39]. This suggests that although attenuated, viruses lacking ubiquitination on K14 may also cause disease in humans. This is also interesting because as new variants appeared causing reduced disease, these mutations on M-K14 appear to dissipate. These observations could be due to either increased immunity in the human population or reduced viral pathogenesis (due to viral adaptation).

## 7. Conclusions and Perspectives

Viruses, including highly pathogenic ones like ZIKV and SARS-CoV-2, rely on host cells’ specific factors for successful replication. Among these factors, the Ub system plays a crucial role. It regulates protein degradation, signal transduction, and DNA repair, and it facilitates virus replication by degrading host factors or modifying viral proteins. Our comprehensive review delves into the complexities of this interaction, with a specific focus on the role of the E3-Ub ligase TRIM7 in the ubiquitination process and its potential implications in viral infection. This analysis underscores the importance of understanding the interplay between viruses and the host cell’s regulatory systems, such as the Ub system, in developing effective host-mediated strategies for combating viral infections.

TRIM7, a protein that ubiquitinates specific structural proteins, is critical in virus replication. This non-degradative mechanism is increasingly recognized as a standard feature of emergent viruses from different families. Recent research in SARS-CoV-2 has unveiled that TRIM7’s ubiquitination of M protein may also have an antiviral function, potentially impeding the spread of the virus. This dual role of TRIM7 underscores the complex nature of the immune response to viral infections. Since ubiquitination sites on the membrane protein of SARS-CoV-2 appeared during the pandemic, it could point to a viral escape mechanism counteracting the antiviral effects of TRIM7, which warrants further investigation.

Our broad understanding of the mechanisms by which viruses replicate, particularly the specific steps involved in ubiquitination, is a crucial step toward effective viral infection management. Future studies should aim to identify the precise subcellular compartments in which this process occurs. This knowledge could pave the way for the development of targeted strategies to disrupt viral replication, a promising avenue in the fight against a wide range of viral infections. These insights hold immense promise, instilling a sense of hope for the future of viral infection management.

## References

[1] Lee J.M., Hammaren H.M., Savitski M.M., Baek S.H. (2023). Control of protein stability by post-translational modifications. Nat. Commun..

[2] Uchil P.D., Hinz A., Siegel S., Coenen-Stass A., Pertel T., Luban J., Mothes W. (2013). TRIM protein-mediated regulation of inflammatory and innate immune signaling and its association with antiretroviral activity. J. Virol..

[3] Komander D., Rape M. (2012). The ubiquitin code. Annu. Rev. Biochem..

[4] van Tol S., Hage A., Giraldo M.I., Bharaj P., Rajsbaum R. (2017). The TRIMendous Role of TRIMs in Virus-Host Interactions. Vaccines.

[5] Damgaard R.B. (2021). The ubiquitin system: From cell signalling to disease biology and new therapeutic opportunities. Cell Death Differ..

[6] Hage A., Rajsbaum R. (2019). To TRIM or not to TRIM: The balance of host-virus interactions mediated by the ubiquitin system. J. Gen. Virol..

[7] Stewart M.D., Ritterhoff T., Klevit R.E., Brzovic P.S. (2016). E2 enzymes: More than just middle men. Cell Res..

[8] Yang Q., Zhao J., Chen D., Wang Y. (2021). E3 ubiquitin ligases: Styles, structures and functions. Mol. Biomed..

[9] Valerdi K.M., Hage A., van Tol S., Rajsbaum R., Giraldo M.I. (2021). The Role of the Host Ubiquitin System in Promoting Replication of Emergent Viruses. Viruses.

[10] Giraldo M.I., Hage A., van Tol S., Rajsbaum R. (2020). TRIM Proteins in Host Defense and Viral Pathogenesis. Curr. Clin. Microbiol. Rep..

[11] Rajsbaum R., Stoye J.P., O’Garra A. (2008). Type I interferon-dependent and -independent expression of tripartite motif proteins in immune cells. Eur. J. Immunol..

[12] Rajsbaum R., Versteeg G.A., Schmid S., Maestre A.M., Belicha-Villanueva A., Martinez-Romero C., Patel J.R., Morrison J., Pisanelli G., Miorin L. (2014). Unanchored K48-linked polyubiquitin synthesized by the E3-ubiquitin ligase TRIM6 stimulates the interferon-IKKepsilon kinase-mediated antiviral response. Immunity.

[13] Versteeg G.A., Rajsbaum R., Sanchez-Aparicio M.T., Maestre A.M., Valdiviezo J., Shi M., Inn K.S., Fernandez-Sesma A., Jung J., Garcia-Sastre A. (2013). The E3-ligase TRIM family of proteins regulates signaling pathways triggered by innate immune pattern-recognition receptors. Immunity.

[14] Di Pietro A., Kajaste-Rudnitski A., Oteiza A., Nicora L., Towers G.J., Mechti N., Vicenzi E. (2013). TRIM22 inhibits influenza A virus infection by targeting the viral nucleoprotein for degradation. J. Virol..

[15] Wang K., Zou C., Wang X., Huang C., Feng T., Pan W., Wu Q., Wang P., Dai J. (2018). Interferon-stimulated TRIM69 interrupts dengue virus replication by ubiquitinating viral nonstructural protein 3. PLoS Pathog..

[16] Wu X., Wang J., Wang S., Wu F., Chen Z., Li C., Cheng G., Qin F.X. (2019). Inhibition of Influenza A Virus Replication by TRIM14 via Its Multifaceted Protein-Protein Interaction With NP. Front. Microbiol..

[17] Bharaj P., Atkins C., Luthra P., Giraldo M.I., Dawes B.E., Miorin L., Johnson J.R., Krogan N.J., Basler C.F., Freiberg A.N. (2017). The Host E3-Ubiquitin Ligase TRIM6 Ubiquitinates the Ebola Virus VP35 Protein and Promotes Virus Replication. J. Virol..

[18] van Tol S., Kalveram B., Ilinykh P.A., Ronk A., Huang K., Aguilera-Aguirre L., Bharaj P., Hage A., Atkins C., Giraldo M.I. (2022). Ubiquitination of Ebola virus VP35 at lysine 309 regulates viral transcription and assembly. PLoS Pathog..

[19] Rodriguez-Salazar C.A., van Tol S., Mailhot O., Gonzalez-Orozco M., Galdino G.T., Warren A.N., Teruel N., Behera P., Afreen K.S., Zhang L. (2024). Ebola virus VP35 interacts non-covalently with ubiquitin chains to promote viral replication. PLoS Biol..

[20] van Gent M., Sparrer K.M.J., Gack M.U. (2018). TRIM Proteins and Their Roles in Antiviral Host Defenses. Annu. Rev. Virol..

[21] Li Y., Wu H., Wu W., Zhuo W., Liu W., Zhang Y., Cheng M., Chen Y.G., Gao N., Yu H. (2014). Structural insights into the TRIM family of ubiquitin E3 ligases. Cell Res..

[22] Skurat A.V., Dietrich A.D., Zhai L., Roach P.J. (2002). GNIP, a novel protein that binds and activates glycogenin, the self-glucosylating initiator of glycogen biosynthesis. J. Biol. Chem..

[23] Zhai L., Dietrich A., Skurat A.V., Roach P.J. (2004). Structure-function analysis of GNIP, the glycogenin-interacting protein. Arch. Biochem. Biophys..

[24] Munoz Sosa C.J., Issoglio F.M., Carrizo M.E. (2021). Crystal structure and mutational analysis of the human TRIM7 B30.2 domain provide insights into the molecular basis of its binding to glycogenin-1. J. Biol. Chem..

[25] Jin J., Lu Z., Wang X., Liu Y., Han T., Wang Y., Wang T., Gan M., Xie C., Wang J. (2020). E3 ubiquitin ligase TRIM7 negatively regulates NF-kappa B signaling pathway by degrading p65 in lung cancer. Cell Signal.

[26] Giraldo M.I., Xia H., Aguilera-Aguirre L., Hage A., van Tol S., Shan C., Xie X., Sturdevant G.L., Robertson S.J., McNally K.L. (2020). Envelope protein ubiquitination drives entry and pathogenesis of Zika virus. Nature.

[27] Lu M., Zhu X., Yang Z., Zhang W., Sun Z., Ji Q., Chen X., Zhu J., Wang C., Nie S. (2019). E3 ubiquitin ligase tripartite motif 7 positively regulates the TLR4-mediated immune response via its E3 ligase domain in macrophages. Mol. Immunol..

[28] Lu M., Ma A., Liu J., Zhou W., Cao P., Chu T., Fan L. (2022). Study on the expression of TRIM7 in peripheral blood mononuclear cells of patients with sepsis and its early diagnostic value. BMC Infect. Dis..

[29] Chakraborty A., Diefenbacher M.E., Mylona A., Kassel O., Behrens A. (2015). The E3 ubiquitin ligase Trim7 mediates c-Jun/AP-1 activation by Ras signalling. Nat. Commun..

[30] Zhou C., Zhang Z., Zhu X., Qian G., Zhou Y., Sun Y., Yu W., Wang J., Lu H., Lin F. (2020). N6-Methyladenosine modification of the TRIM7 positively regulates tumorigenesis and chemoresistance in osteosarcoma through ubiquitination of BRMS1. EBioMedicine.

[31] Zhou F., Liu Y., Ai W., Wang Y., Gan M., Jiang Q., Han T., Wang J.B. (2022). GNIP1 functions both as a scaffold protein and an E3 ubiquitin ligase to regulate autophagy in lung cancer. Cell Commun. Signal.

[32] Chen Q., Zhang T., Zeng R., Zhang K., Li B., Zhu Z., Ma X., Zhang Y., Li L., Zhu J. (2024). The E3 ligase TRIM7 suppresses the tumorigenesis of gastric cancer by targeting SLC7A11. Sci. Rep..

[33] Zhu L., Qin C., Li T., Ma X., Qiu Y., Lin Y., Ma D., Qin Z., Sun C., Shen X. (2020). The E3 ubiquitin ligase TRIM7 suppressed hepatocellular carcinoma progression by directly targeting Src protein. Cell Death Differ..

[34] Wang J., Qin X., Huang Y., Zhang Q., Pei J., Wang Y., Goren I., Ma S., Song Z., Liu Y. (2023). TRIM7/RNF90 promotes autophagy via regulation of ATG7 ubiquitination during *L. monocytogenes* infection. Autophagy.

[35] Li D., Wu M. (2021). Pattern recognition receptors in health and diseases. Signal Transduct. Target. Ther..

[36] Thompson M.R., Kaminski J.J., Kurt-Jones E.A., Fitzgerald K.A. (2011). Pattern recognition receptors and the innate immune response to viral infection. Viruses.

[37] Yang B., Zhang G., Qin X., Huang Y., Ren X., Sun J., Ma S., Liu Y., Song D., Liu Y. (2021). Negative Regulation of RNF90 on RNA Virus-Triggered Antiviral Immune Responses Targeting MAVS. Front. Immunol..

[38] Yang B., Liu Y., Cui Y., Song D., Zhang G., Ma S., Liu Y., Chen M., Chen F., Wang H. (2020). RNF90 negatively regulates cellular antiviral responses by targeting MITA for degradation. PLoS Pathog..

[39] Gonzalez-Orozco M., Tseng H.C., Hage A., Xia H., Behera P., Afreen K., Penaflor-Tellez Y., Giraldo M.I., Huante M., Puebla-Clark L. (2024). TRIM7 ubiquitinates SARS-CoV-2 membrane protein to limit apoptosis and viral replication. bioRxiv.

[40] Li M., Yan J., Zhu H., Guo C., Jiang X., Gao Y., Liu X., Jiang P., Bai J. (2023). TRIM7 inhibits encephalomyocarditis virus replication by activating interferon-beta signaling pathway. Vet. Microbiol..

[41] Ma X., Yuan Z., Yi Z. (2022). Identification and characterization of key residues in Zika virus envelope protein for virus assembly and entry. Emerg. Microbes Infect..

[42] Fan W., Mar K.B., Sari L., Gaszek I.K., Cheng Q., Evers B.M., Shelton J.M., Wight-Carter M., Siegwart D.J., Lin M.M. (2021). TRIM7 inhibits enterovirus replication and promotes emergence of a viral variant with increased pathogenicity. Cell.

[43] Baggen J., Thibaut H.J., Strating J., van Kuppeveld F.J.M. (2018). The life cycle of non-polio enteroviruses and how to target it. Nat. Rev. Microbiol..

[44] Liang X., Xiao J., Li X., Liu Y., Lu Y., Wen Y., Li Z., Che X., Ma Y., Zhang X. (2022). A C-terminal glutamine recognition mechanism revealed by E3 ligase TRIM7 structures. Nat. Chem. Biol..

[45] Ru Y., Yan X., Zhang B., Song L., Feng Q., Ye C., Zhou Z., Yang Z., Li Y., Zhang Z. (2022). C-terminal glutamine acts as a C-degron targeted by E3 ubiquitin ligase TRIM7. Proc. Natl. Acad. Sci. USA.

[46] Luptak J., Mallery D.L., Jahun A.S., Albecka A., Clift D., Ather O., Slodkowicz G., Goodfellow I., James L.C. (2022). TRIM7 Restricts Coxsackievirus and Norovirus Infection by Detecting the C-Terminal Glutamine Generated by 3C Protease Processing. Viruses.

[47] Orchard R.C., Sullender M.E., Dunlap B.F., Balce D.R., Doench J.G., Virgin H.W. (2019). Identification of Antinorovirus Genes in Human Cells Using Genome-Wide CRISPR Activation Screening. J. Virol..

[48] Sullender M.E., Pierce L.R., Annaswamy Srinivas M., Crockett S.L., Dunlap B.F., Rodgers R., Schriefer L.A., Kennedy E.A., Stewart B.M., Doench J.G. (2022). Selective Polyprotein Processing Determines Norovirus Sensitivity to Trim7. J. Virol..

[49] Fan W., McDougal M.B., Schoggins J.W. (2022). Enterovirus 3C Protease Cleaves TRIM7 To Dampen Its Antiviral Activity. J. Virol..

[50] Chen J., Li Z., Guo J., Xu S., Zhou J., Chen Q., Tong X., Wang D., Peng G., Fang L. (2022). SARS-CoV-2 nsp5 Exhibits Stronger Catalytic Activity and Interferon Antagonism than Its SARS-CoV Ortholog. J. Virol..

[51] Liang W., Gu M., Zhu L., Yan Z., Schenten D., Herrick S., Li H., Samrat S.K., Zhu J., Chen Y. (2023). The main protease of SARS-CoV-2 downregulates innate immunity via a translational repression. Signal Transduct. Target. Ther..

[52] Shemesh M., Aktepe T.E., Deerain J.M., McAuley J.L., Audsley M.D., David C.T., Purcell D.F.J., Urin V., Hartmann R., Moseley G.W. (2021). SARS-CoV-2 suppresses IFNbeta production mediated by NSP1, 5, 6, 15, ORF6 and ORF7b but does not suppress the effects of added interferon. PLoS Pathog..

[53] Zheng Y., Deng J., Han L., Zhuang M.W., Xu Y., Zhang J., Nan M.L., Xiao Y., Zhan P., Liu X. (2022). SARS-CoV-2 NSP5 and N protein counteract the RIG-I signaling pathway by suppressing the formation of stress granules. Signal Transduct. Target. Ther..

[54] Liu Y., Qin C., Rao Y., Ngo C., Feng J.J., Zhao J., Zhang S., Wang T.Y., Carriere J., Savas A.C. (2021). SARS-CoV-2 Nsp5 Demonstrates Two Distinct Mechanisms Targeting RIG-I and MAVS To Evade the Innate Immune Response. mBio.

[55] Stukalov A., Girault V., Grass V., Karayel O., Bergant V., Urban C., Haas D.A., Huang Y., Oubraham L., Wang A. (2021). Multilevel proteomics reveals host perturbations by SARS-CoV-2 and SARS-CoV. Nature.

[56] Yuan Z., Hu B., Xiao H., Tan X., Li Y., Tang K., Zhang Y., Cai K., Ding B. (2021). The E3 Ubiquitin Ligase RNF5 Facilitates SARS-CoV-2 Membrane Protein-Mediated Virion Release. mBio.

[57] Chu H., Hou Y., Yang D., Wen L., Shuai H., Yoon C., Shi J., Chai Y., Yuen T.T., Hu B. (2022). Coronaviruses exploit a host cysteine-aspartic protease for replication. Nature.

